# Traditional Chinese medicine for knee osteoarthritis: An overview of systematic review

**DOI:** 10.1371/journal.pone.0189884

**Published:** 2017-12-21

**Authors:** Min Yang, Li Jiang, Qing Wang, Hao Chen, Guihua Xu

**Affiliations:** 1 School of Nursing, Nanjing University of Chinese Medicine, Nanjing, Jiangsu, China; 2 Jingjiang College, Jiangsu University, Zhenjiang, Jiangsu, China; 3 The Second Clinical Medical School, Nanjing University of Chinese Medicine, Nanjing, Jiangsu, China; Stanford University School of Medicine, UNITED STATES

## Abstract

**Background:**

Traditional Chinese medicine (TCM) has been accepted as a complementary therapy for knee osteoarthritis. However, the efficacy and safety of the intervention were still conflicting and uncertain. Meanwhile, the quality of methodology and evidence in the field was unknown.

**Objective:**

To summarize the characteristics and critically evaluate the quality of methodology, as well as the evidence of systematic reviews (SRs) on TCM for knee osteoarthritis.

**Methods:**

Five electronic databases were searched from inception to April 2016. The methodological quality of the included studies was assessed by AMSTAR and ROBIS. The quality of the evidence was determined using the GRADE approach.

**Results:**

Ten SRs were included. The conclusions suggest that TCM provides potential benefits for patients with knee osteoarthritis. These benefits include pain relief, functional improvement, and presence of few adverse events. Limitations of the methodological quality mainly included the lack of a-priori protocol or protocol registration and incomprehensive literature search. A list of excluded studies was also not provided. The overall quality of evidence in the SRs was poor, ranging from “very low” to “low,” mainly because of the serious risk of bias of original trials, inconsistencies, and imprecision in the outcomes.

**Conclusions:**

TCM generally appears to be effective for knee osteoarthritis treatment. However, the evidence is not robust enough because of the methodological flaws in SRs. Hence, these conclusions on available SRs should be treated with caution for clinical practice.

## Introduction

Knee osteoarthritis (OA) is a common joint disease worldwide and is the leading cause of pain and disability in the elderly. In the first decade of the 21st century, nearly 27 million Americans had suffered from symptomatic knee OA, with advanced OA accounting more than half of them [1]. The national prevalence rate of knee OA in the adult Portuguese was 12.4% (11.0% to 13.8%) [2]. In addition, a longitudinal study involving 17,128 Chinese residents aged 45 years and older showed that the overall prevalence of symptomatic knee OA was 8.1% [3]. In rural China, symptomatic knee OA was more common (16.57%), and this incidence increased significantly in people aged 70 years and older (29.25% for women and 24.71% for men) [4]. Patients with knee OA usually experience chronic pain and physical limitation, as well as psychiatric disorders [5], consequently affecting the patients’ quality of life in various levels [6]. The number of people living with knee OA is expected to increase because of the global aging population and obesity [7]; thus, the burden of knee OA on patients will increase without effective treatments for symptoms. Therefore, interventions intended to relieve the pain and enhance the mobility, functionality, and quality of life should be developed to improve the management of knee OA. Traditional Chinese medicine (TCM) has been accepted as a complementary therapy for knee OA, not only in Asian countries [8, 9] but also in the West [10, 11] which might result from its effects on pain, loss of mobility and function as well as depression [12]. Currently, the use of TCM, such as acupuncture and Tai Chi, has been included in the OARSI guidelines [13] and advocated by reviews [14] for non-surgical management of knee OA.

Systematic reviews (SRs) are the source of high-quality evidence, which can provide appropriate conclusions for making clinical decisions. However, only high-quality SRs are reliable because low-quality SRs may reduce the value of the results or even mislead the clinical decision [15]. Despite the increase in publication of SRs regarding TCM in knee OA, the evidence from these SRs has not been assessed systematically. The overview of SRs is an approach to compile the evidence and synthesize the results from multiple SRs into one accessible document [16], providing a strategic direction to implement future SRs. Furthermore, an overview facilitates the discovery of potential “evidence gaps”, thus informing new SRs where to give priority. The present overview aims to summarize the characteristics and critically evaluate the quality of methodology, as well as the evidence of SRs on TCM for the treatment of knee OA, thus providing a comprehensive “user-friendly front end” for clinical practitioners and researchers.

## Methods

### Criteria for considering SRs for inclusion

In this overview, we considered SRs containing at least one randomized controlled trial (RCT), which addressed the treatment of TCM in knee OA. More specifically, we used the PICO inclusion criteria: participants, interventions, comparisons, and outcomes.

#### Participants

Patients diagnosed with knee OA, at any severity, which were either (1) diagnosed by the American College of Rheumatology (ACR) criteria, (2) Chinese Medical Association criteria, (3) or European League against Rheumatism (EULAR) criteria, were included.

#### Interventions

All types of interventions pertaining to TCM were considered, and interventions can include (but not be limited to) the following: acupuncture, electroacupuncture, Chinese herbal treatment, moxibustion, Tai Chi, Qigong, Chinese herbal bath, and massage.

#### Comparisons

The control interventions included non-treatment, sham treatment, placebo treatment, and routine treatments (e.g., health education, weight management, strength training, physiotherapy, acetaminophen therapy, glucosamine treatment, and NSAID therapy).

#### Outcomes

In accordance with the Osteoarthritis Research Society International (OARSI) Clinical Trials Recommendations [17], we categorized the outcomes into seven types: binary benefit outcomes, patient-reported outcomes, objective outcomes, structural outcomes, biochemical biomarkers, adverse effects, and economic evaluations. Among these categories, the binary benefit outcomes included all-cause mortality, recovery, and disability events. The core set patient-reported outcomes were assessed using three clinical measures, namely, pain, physical function, and patients’ global assessment, which were specified in the Outcome Measures in Rheumatology Clinical Trials (OMERACT) III Conference [18]. All the binary benefit outcomes were regarded as the primary outcomes, whereas the remaining six outcomes were considered secondary.

### Search methods for identification of SRs

Five electronic databases [PubMed, Medline, PsycINFO (OvidSP), Embase (OvidSP), and Cochrane Library] were searched from inception to April 2016. Intervention search terms were not included in the search terms because we wish to identify all SRs for any types of TCM, and only terms of knee OA and publication type were incorporated. The search language was restricted to English. The literature search was composed of MeSH terms and free-text words for “knee osteoarthritis”, “systematic review”, and “meta-analysis”, which were adopted for different databases. For example, the search strategy on the PubMed database was documented as follows: (Osteoarthritis, knee [Mesh Terms] OR knee osteoarthritides [Title/Abstract] OR knee osteoarthritis [Title/Abstract] OR osteoarthritides, knee [Title/Abstract] OR knee, osteoarthritis of [Title/Abstract] OR knees, osteoarthritis of [Title/Abstract] OR osteoarthritis of knee [Title/Abstract]) AND (Review [Publication Type] OR systematic review [Title/Abstract] OR meta-analysis [Title/Abstract] OR Meta-Analysis [Publication Type] OR Meta-Analysis as Topic [Mesh Terms]). Additionally, the reference lists of the included articles were hand-searched for relevant articles.

### Selection of SRs

Initially, the titles and abstracts of the identified articles were reviewed. Full-text articles of all potentially included studies were checked to determine their eligibility. Two authors (MY and LJ) independently applied the above criteria for selection of SRs. Any disagreement was resolved by discussion or judged by the third author (QW) if a consensus was not reached.

### Data extraction

Two authors (MY and LJ) independently extracted data from the reviews and on completion of extraction, as well as cross-checked each other’s extracted data. Discrepancies were resolved via consensus with a third author. If the information described in SRs was unclear or omitted, we accessed the primary trials. We extracted the following data from the included SRs: author/s, publication year, sample size, diagnostic criteria, patient’s age, duration and severity of knee OA, details of intervention (including types, medication doses, and treatment duration), controlled regimen, and outcomes. We contacted the corresponding authors of the SRs or original trials if the essential information was inadequate from the reports.

### Assessment of methodological quality

Two authors (MY and LJ) evaluated the methodological quality of the included SRs by using two assessment tools: a measurement tool to assess the methodological quality of systematic reviews (AMSTAR) [19] and the Risk of Bias in Systematic Review (ROBIS) [20]. AMSTAR is a tool developed to measure how SRs avoided or reduced the risk of bias; this tool was demonstrated to be relatively easy, reliable, and valid for the methodological quality assessment of SRs on TCM [21]. The checklist consists of 11 questions answerable by a yes, no, or unclear or not applicable responses. ROBIS, a new tool used to assess the risk of bias during the process of design, conduct, and analysis of SRs, is completed in three phases: assessing relevance between the target question and question of SR, identifying concerns with the SR process, and judging the risk of bias. Answers to the signal questions in ROBIS can be categorized as yes, probably yes, no, probably no, or no information. The risk of bias in the SR was judged as low, high, or unclear. Two authors (MY and LJ) extracted the data independently, and any inconformity was resolved by discussing and making consensus with a third author (HC). The kappa statistics [22] was calculated to understand the extent of interobserver agreement in terms of AMSTAR and ROBIS items. Kappa less than 0.2 is defined as ‘poor agreement’, 0.2 to 0.4 as ‘fair agreement’, 0.4 to 0.6 as ‘moderate agreement’, 0.6 to 0.8 as ‘substantial agreement’, and a kappa = 0.8 to 1.0 as ‘almost perfect agreement’.

### Assessment of quality of evidence

The quality of evidence of the included SRs was determined using the Grading of Recommendations Assessment, Development and Evaluation (GRADE) approach. This tool is designed to evaluate the quality of evidence for each outcome across studies (also called a body of evidence) [23]. Two authors (MY and GHX) independently assessed the evidence pertaining to outcomes, and the upgraded or downgraded factors affecting the quality of evidence should be depicted in detail to guarantee the transparency and reliability of the results. The factors were related to the risk of bias, inconsistency, indirectness, precision, and publication bias. The overall quality of evidence was judged as high, moderate, low, or very low.

### Data analysis

The narrative summary of the characteristics of the included SRs is displayed in tables. The dichotomous data were summarized as the odds ratio (OR) or risk ratio (RR), and continuous outcomes were synthesized as weighted or standard mean difference (WMD/SMD), with 95% confidence intervals (CIs). For data that were very heterogeneous to pool or presented in format like medians, which were unsuitable for pooling, we employed a narrative synthesis. For categorical variables, we presented frequencies with percentages as appropriate, and for continuous variables, we reported them as SMD or medians with interquartile ranges (IQRs).

## Results

### Selection of studies

**Fig 1:**
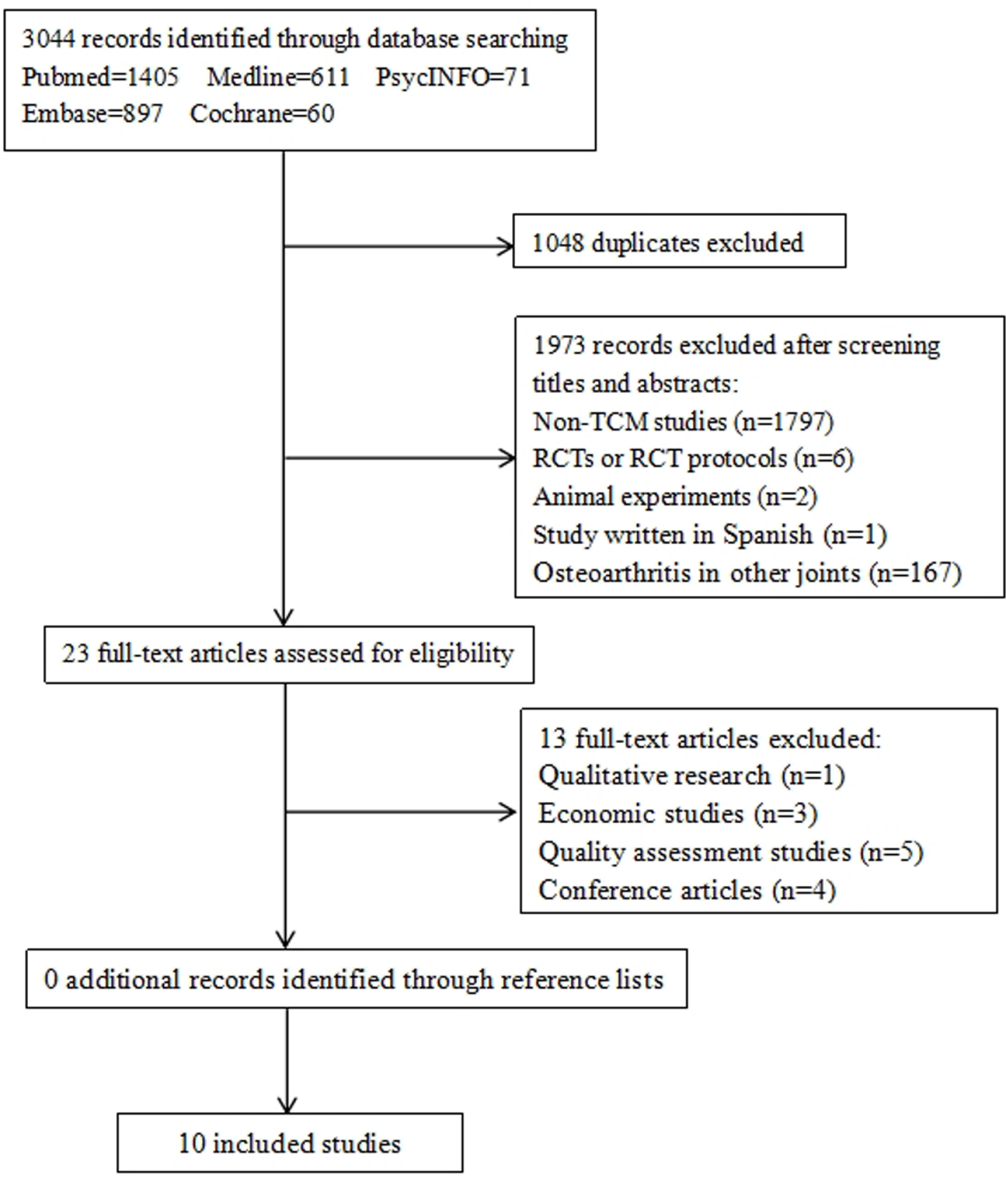
Flow diagram of literature retrieval and selection.

Initially, a total of 3044 potentially relevant articles were identified in the literature search. Citations for all the articles were imported into Endnote software, and duplicates (n = 1048) were filtered automatically. After screening the titles and abstracts, 1797 non-TCM studies, 6 RCTs or RCT protocols, 2 animal experiments, 1 study written in Spanish, and 167 studies involving OA in other joints were excluded. Subsequently, full texts of the remaining articles were reviewed, and 1 qualitative research, 3 economic studies, and 5 quality assessment studies were removed. Four conference articles, which were reported only in the format of abstracts and tables, were excluded. We did not identify additional studies via a hand search of relevant references. Finally, 10 SRs [14, 24–32] on TCM for knee OA met the inclusion criteria (Fig 1).

### Characteristics of included SRs

The main characteristics of the 10 included SRs are summarized in Table 1. The 10 SRs involved five specific TCM techniques (acupuncture [24–26], moxibustion [27, 28] Chinese herbal medicine [29, 30] Chinese herbal bath [31], and Tai Chi [32]) and one SR that reviewed the general efficacy of TCM [14], including acupuncture, Qigong, and herbs. All the SRs were published within the last 10 years, with the oldest study being from 2006 [26], and were conducted by a median of 5.5 authors (range: 4 to 7). The 10 included SRs covered 96 RCTs, of which sample size, characteristics of patients, interventions, and primary outcomes are reported in S1 Table.

**Table 1:** Characteristics of included systematic reviews (n = 10).

Study ID	Included Trials and Participants(n)	Interventions		Quality of original trials (Quality assessment tool)	Main Results
		Treatment group	Control group		
Hou, 2015^[^^14^^]^	18 (3023)	Acupuncture; Chinese medicine; qigong	Education; sham acupuncture; placebo; physiotherapy; exercise and advice; drug therapy; no treatment	High quality 10, moderate quality 6, low quality 2 (Cochrane Back Review Group criteria)	Acupuncture is a promising intervention for curing pain, and qigong with motion is an effective method for treating physical function (descriptive summary).
Cao, 2012^[^^24^^]^	14 (3835)	Acupuncture	Sham acupuncture; usual care; waiting list	High quality 11, low quality 3 (Cochrane Back Review Group criteria)	(1) Compared with sham acupuncture, acupuncture was better at relieving pain(SMD -0.25 [95% CI, -0.42 to -0.09]) and restoring function (SMD -0.22 [95% CI, -0.40 to -0.05]) in the short-term period, and relieving pain (SMD -0.10 [95% CI, -0.21 to -0.01]) and restoring function (SMD -0.11 [95% CI, -0.22 to -0.00]) in the long-term. (2) Compared with the standard care,acupuncture was better at relieving pain (SMD -0.43 [95% CI, -0.63 to -0.23]) and restoring function (SMD -0.36 [95% CI, -0.54 to -0.18]), and relieving pain (SMD -0.35 [95% CI, -0.63 to -0.07]) and restoring function (SMD-0.29 [95% CI, -0.53 to -0.05]) in the long-term. (3) Compared with the waiting list,acupuncture was better at relieving pain (SMD -0.89 [95% CI, -1.10 to -0.67]) and restoring function (SMD-0.83 [95% CI, -1.08 to -0.58]).
Manheimer, 2007^[^^25^^]^	11 (2821)	Acupuncture	Sham acupuncture; waiting list; physician visits with consultation and prescription for diclofenac	Score: 4–10 (Cochrane Back Review Group criteria)	(1) Compared with the waiting list, acupuncture improved pain (SMD -0.96 [95% CI, -1.21 to -0.70]) and function (SMD -0.93 [95% CI, -1.16 to -0.69]) in the short-term. (2) Compared with the usual care, acupuncture also improved pain (SMD -0.62, [95% CI, -0.75 to -0.49]) and function (SMD -0.56, [95% CI, -0.69 to -0.43]) in the short- and long-term. (3) Compared with a sham control, acupuncture provided clinically irrelevant short-term improvements in pain (SMD-0.35 [95% CI, -0.55 to -0.15]) and function (SMD-0.35 [95% CI, -0.56 to -0.14]) and clinically irrelevant long-term improvements in pain (SMD-0.13 [95%CI, -0.24 to -0.01]) and function (SMD-0.14 [95% CI, -0.26 to -0.03]).
Yamashita, 2006^[^^26^^]^	7 (4588)	Acupuncture	Sham acupuncture; no treatment	Not reported	Many adverse reactions to acupuncture treatment reported in RCTs, at least for the knee OA, are non-specific, and that not all reported events should be attributed to the mechanism of action of acupuncture.
Li, 2016^[^^27^^]^	4 (746)	Moxibustion	Sham moxibustion; usual care; drug therapy	Low or moderate quality (Cochrane risk of bias)	(1) In terms of quality of life (QOL), moxibustion only had effects in body pain (BP) compared with those in the control group (WMD4.36 [95%CI, 2.27 to 6.44]) in all of the subcategories of the SF-36 scale. (2) There was not a statistically significant difference in the pain or function subscale for the WOMAC scale (WMD 17.63 [95% CI,23.15 to 58.41]).
Song, 2016^[^^28^^]^	13 (1615)	Moxibustion	Sham moxibustion; usual care; drug therapy	/ (Cochrane risk of bias)	(1) Moxibustion is not statistically different from oral drug in improving the response rate (RR 1.09 [95% CI, 1.00 to 1.20]), alleviating pain and improving physical function. (2) Moxibustion is superior to usual care and sham moxibustion in reducing WOMAC score (MD 7.56 [95% CI, 4.11 to 11.00]), pain and function, as well as increasing quality of life.
Zhang, 2016^[^^29^^]^	12 (982)	DJD; DJD plus interventions in control group	Drug therapy	High risk of bias (Cochrane risk of bias)	(1) DJD combined with glucosamine (MD 4.20 [95% CI, 1.72 to 6.69]); or DJD plus meloxicam and glucosamine (MD 3.48 [95%CI 1.59to 5.37])improved total WOMAC scores. (2) DJD plus sodium hyaluronate injection improved pain(MD 0.89 [95% CI, 0.26 to 1.53]).
Zhu, 2015^[^^30^^]^	26 (11277)	MCHF	Drug therapy; usual care; intra-articular injection therapy	Most were high risk of bias (Cochrane risk of bias)	(1) MCHF significantly relieved the global pain of knee joints (MD 0.73 [95% CI, 0.20 to 1.26]). (2) MCHF plus routine treatments significantly decreased the scores of WOMAC (MD 1.16 [95% CI, 0.82 to 1.49]) and Lequesne index (MD 1.49 [95% CI, 0.01 to 2.96]). (3) There were no statistical differences between MCHF group and routine treatment group in walk-related pain (MD 0.24 [95% CI, −0.18 to 0.66]) and WOMAC scores (MD 0.06 [95% CI, −0.39 to 0.51]). No significant differences were found in Lysholm scores (MD 5.10 [95% CI, −3.21 to 13.42]), (MD 5.30 [95% CI, −2.96 to 13.56]).
Chen, 2015^[^^31^^]^	14 (1618)	Chinese herbal bath	Drug therapy	Moderate (Newcastle-Ottawa Scale)	Chinese herbal bath improved pain (MD −0.59 [95% CI, −0.83 to−0.36]) and total effectiveness rate (RR 1.21 [95% CI, 1.15 to 1.28]) compared with standard western treatment.
Ye, 2014^[^^32^^]^	6 (314)	Tai Chi	Education; usual care; no treatment	Moderate (Physiotherapy Evidence Database (PEDro) scale)	Tai Chi was an effective way of relieving pain and improving physical function(descriptive summary).

**Notes:** DJD, Duhuo Jisheng decoction; MCHF, Manufactured Chinese herbal formula.

All SRs contained only RCTs. The SRs included a median of 12.5 trials (range: 4 to 26), involving a total of 20473 participants, and each SR contained a median of 2219.5 participants (range: 314 to 11277). Three SRs [14, 26, 32] (3/10, 30%) did not conduct a meta-analysis because of the heterogeneity of study design and treatment style.

For outcomes, no SR had reported binary benefit outcomes, structural outcomes, biochemical biomarkers, and economic evaluations. Only two SRs [27, 28] considered the quality of life (2/10, 20%). Most of the SRs assessed adverse effects [25, 26, 28–31] (6/10, 60%) and symptoms [14, 24, 25, 27, 28, 29, 30, 31, 32] (9/10, 90%). Specifically, nine SRs [14, 24, 25, 27, 28, 29, 30, 31, 32] reported pain (9/10, 90%), eight [14, 24, 25, 27, 28, 30, 32] assessed physical function (7/10, 70%), and one SR [31] measured the effective rate (1/10, 10%).

As presented in Table 1, most of the SRs showed that TCM can improve the quality of life of patients diagnosed with knee OA (2/2, 100%) and provide potential benefits in alleviating pain (8/9, 88.9%) and improving physical function (6/7, 85.7%). For adverse effects, none of the SRs had pooled the adverse events and conducted the forest plot because of the limited reporting and heterogeneous methods. No serious adverse reactions associated with TCM were reported. One SR [26] focused on specific reactions to acupuncture, and this SR confirmed that not all reported adverse events should be attributed to the mechanism of acupuncture.

### Methodological quality of the included SRs

#### Methodological quality of included SRs assessed by AMSTAR

The quality of the included SRs was low to moderate, as determined using the AMSTAR tool (Table 2). None of the SRs registered a protocol or provided a list of excluded studies. Four SRs [24, 28–30] (4/10, 40%) performed a comprehensive literature search. The remaining SRs were judged as “No” for this item because of the following reasons: one SR [14] considered only one database; two SRs [25, 27] did not provide concrete key words and/or MESH terms; searches in three SRs [26, 31, 32] were not supplemented by textbooks, specialized registers, or references in the studies found. Five SRs [24, 25, 28, 30, 31] (5/10, 50%) stated no restriction to the language or publication type when excluding the reports, and the language of one SR [27] (1/10, 10%) was restricted to English.

**Table 2:** Assessment of methodological quality using AMSTAR tool.

Appraisal criteria	Hou, 2015^[^^14^^]^	Cao, 2012^[^^24^^]^	Manheimer, 2007^[^^25^^]^	Yamashita, 2006^[^^26^^]^	Li, 2006^[^^27^^]^	Song, 2006^[^^28^^]^	Zhang, 2016^[^^29^^]^	Zhu, 2015^[^^30^^]^	Chen, 2015^[^^31^^]^	Ye, 2014^[^^32^^]^
1. Was an ‘a priori’ design provided?	N	N	N	N	N	N	N	N	N	N
2. Was there duplicate study selection and data extraction?	N	Y	Y	N	Y	Y	Y	Y	Y	N
3. Was a comprehensive literature search performed?	N	Y	N	N	N	Y	Y	Y	N	N
4. Was the status of publication (i.e. grey literature) used as an inclusion criterion?	N	Y	Y	N	Y	Y	N	Y	Y	N
5. Was a list of studies (included and excluded) provided?	N	N	N	N	N	N	N	N	N	N
6. Were the characteristics of the included studies provided?	Y	Y	Y	Y	Y	Y	Y	Y	Y	Y
7. Was the scientific quality of the included studies assessed and documented?	Y	Y	Y	N	Y	Y	Y	Y	Y	Y
8. Was the scientific quality of the included studies used appropriately in formulating conclusion?	Y	Y	Y	N	Y	Y	Y	Y	Y	Y
9. Were the methods used to combine the findings of studies appropriately?	Y	Y	Y	Y	N	Y	Y	N	Y	Y
10. Was the likelihood of publication bias assessed?	Y	N	Y	N	Y	Y	Y	Y	Y	N
11. Was the conflict of interest stated?	Y	N	Y	Y	Y	N	Y	Y	Y	Y

**Notes:** Y: Yes; N: No.

The characteristics of the included studies were presented in all SRs (10/10, 100%). Although one SR [26] neither assessed the quality of included studies nor formulated a conclusion appropriately, the conclusions of the nine other SRs (9/10, 90%) drawn from included studies were prudent and rigorous in terms of quality. Eight SRs (8/10, 80%) used appropriate methods to combine the findings, whereas one SR [27] pooled data without considering the distinction of control treatment, and another SR [30] did not analyze the source of heterogeneity, despite facing a high *P* value of heterogeneity test (*P*< 0.10, I^2^ = 99%). Publication bias was assessed in two SRs [25, 30] (2/10, 20%) via funnel plot. Furthermore, three SRs stated that funnel plot analysis cannot be completed because of the unanimous publication or the small number of included trials [28, 29, 31] and two SRs omitted the publication bias because of the language restriction [14, 27]. Eight SRs [14, 25–27, 29–32] (8/10, 80%) stated a conflict of interest.

#### Risk of bias of the included SRs assessed by ROBIS

**Fig 2:**
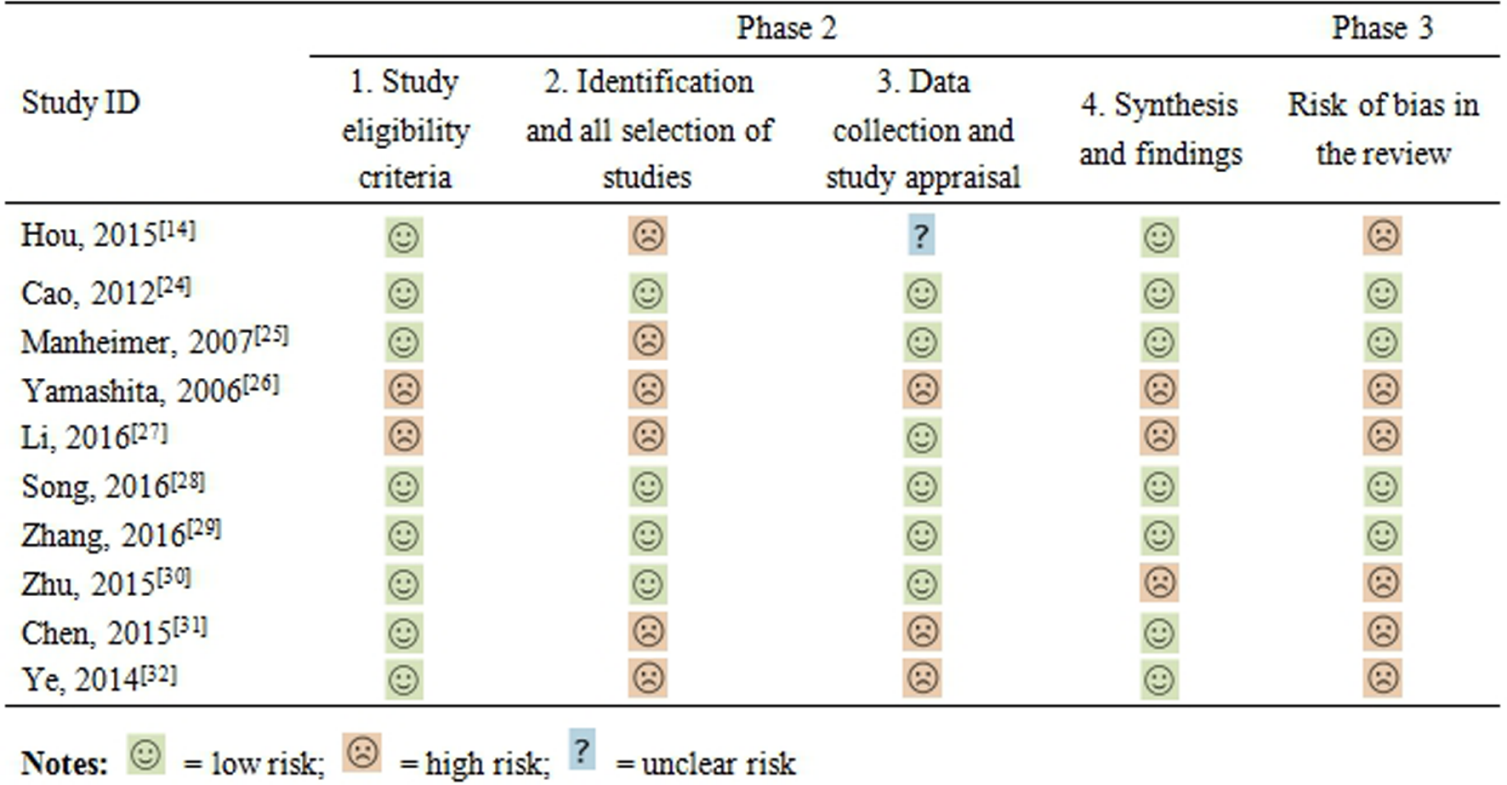
Assessment of methodological quality using ROBIS tool.

**Fig 3:**
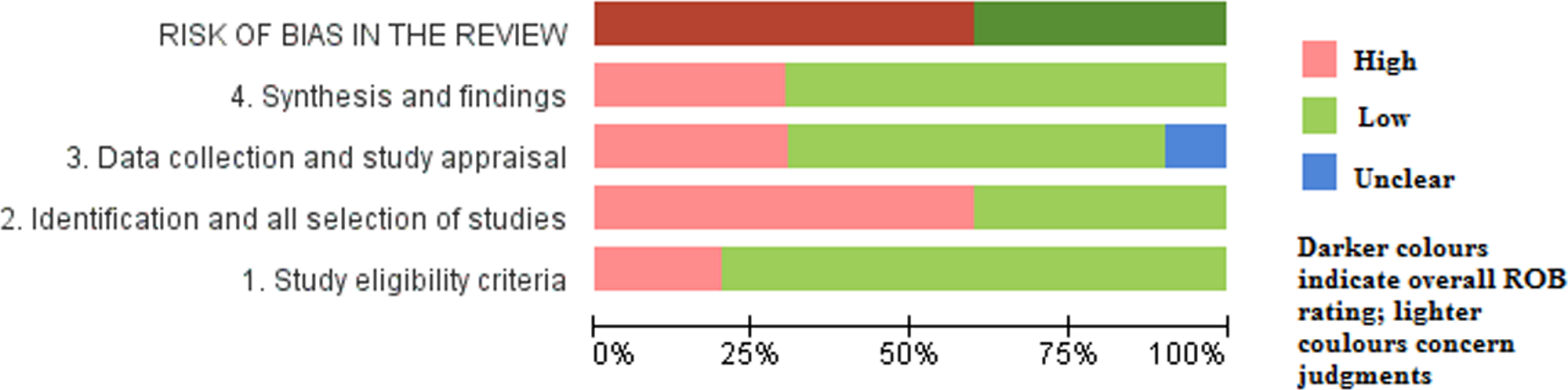
Risk of bias of included systematic reviews assessed by ROBIS.

The risk of bias of included SRs was assessed by ROBIS, as displayed in Fig 2. For the first domain of phase 2, the eligibility criteria of the participants in two SRs [27, 32] were unambiguous. For the second domain, in six SRs [14, 25–27, 31, 32] added to some databases, sources like conferences or trial records were not searched, or the suitable subject indexing was not reported. For the third domain, the risk of bias in one SR [14] was unclear because it did not report the duplicate-data extraction (or single-data extraction with rigorous checking), which is necessary to safeguard against random errors. Additionally, the evaluation criteria in two SRs [31, 32] were thought to be insufficient to identify all potential biases in primary studies, and one SR [26] did not evaluate the bias of the included studies. For the fourth domain, the risk of bias in three SRs was judged as high. One SR [27] used pooling data without considering the clinical heterogeneity, one SR [30] did not address the heterogeneity, and one SR [26] ignored the bias of primary studies. Unfortunately, the risk of bias in previous domains was interpreted appropriately in none of the above SRs. Therefore, the risk of bias in the six SRs mentioned previously was high, whereas that in the other SRs was low. Considering the number of included trials in SRs, as well as the total number of participants, the risk of bias is summarized in Fig 3.

The kappa value showed that the consistency of subjective evaluation of two reviewers (MY and LJ) in terms of AMSTAR (0.874) and ROBIS (0.901) items was good.

### Quality of evidence in the included SRs assessed by GRADE

The quality of evidence for 20 main outcomes in 10 included SRs is presented in Table 3. By using the GRADE approach, high or moderate quality of evidence was found in none of the 10 included SRs. The evidence was downgraded to either “low” or “very low” quality because of the following limitations: (1) Most of the original RCTs were of poor quality. The bias of blinding, allocation concealment, and intention to treat analysis decreased the validity of the GRADE approach. (2) In one study, inconsistencies were noted for pooling data pertaining to different control interventions (downgraded by two levels). The remaining majority outcomes were downgraded by one level because of differences in intervention details and methodological quality. (3) For nearly half of the main outcomes, owing to the small number of participants (<400), as well as the wide CIs or inappreciable benefits, we subsequently downgraded their quality of evidence based on imprecision. (4) The high probability of publication bias cannot be ruled out because of the incomprehensive literature search, as well as the predominance of favorable outcomes. The more detailed information regarding the reason for downgrading of each outcome was presented in S2 Table.

**Table 3:** Summary of findings table: Quality of evidence in included SRs assessed by GRADE.

Intervention	Control	Main outcome	Quality of evidence	Study ID
Acupuncture	Sham acupuncture	Pain relief	Low	Hou, 2015^[^^14^^]^; Cao, 2012^[^^24^^]^; Manheimer, 2007^[^^25^^]^
		Function improving	Low	Cao, 2012^[^^24^^]^; Manheimer, 2007^[^^25^^]^
		Function improving	Very low	Hou, 2015^[^^14^^]^
		Less adverse effects	Very low	Yamashita, 2006^[^^24^^]^
	Waiting list	Pain relief	Very low	Cao, 2012^[^^24^^]^; Manheimer, 2007^[^^25^^]^
		Function improving	Very low	Hou, 2015^[^^14^^]^; Cao, 2012^[^^24^^]^; Manheimer, 2007^[^^25^^]^
	Usual care	Pain relief	Very low	Cao, 2012^[^^24^^]^; Manheimer, 2007^[^^25^^]^
		Function improving	Very low	Hou, 2015^[^^14^^]^; Cao, 2012^[^^24^^]^; Manheimer, 2007^[^^25^^]^
	Education	Pain relief	Low	Hou, 2015^[^^14^^]^
		Function improving	Very low	Hou, 2015^[^^14^^]^
	Physiotherapy	Pain relief	Low	Hou, 2015^[^^14^^]^
		Function improving	Very low	Hou, 2015^[^^14^^]^
	Exercise	Pain relief	Low	Hou, 2015^[^^14^^]^
		Function improving	Very low	Hou, 2015^[^^14^^]^
	No treatment	Less adverse effects	Very low	Yamashita, 2006^[^^26^^]^
Moxibustion	Sham moxibustion	Quality of life (BP)	Low	Li, 2016^[^^27^^]^
		Pain relief	Very low	Li, 2016^[^^27^^]^; Song, 2016^[^^28^^]^
		Function improving	Very low	Li, 2016^[^^27^^]^; Song, 2016^[^^28^^]^
	Usual care	Pain relief	Very low	Li, 2016^[^^27^^]^; Song, 2016^[^^28^^]^
		Function improving	Very low	Li, 2016^[^^27^^]^; Song, 2016^[^^28^^]^
	Drug therapy	Pain relief	Very low	Li, 2016^[^^27^^]^
		Function improving	Very low	Li, 2016^[^^27^^]^
	Intra-articular injection	Response rate improving	Low	Song, 2016^[^^28^^]^
	Oral drug	Response rate improving	Low	Song, 2016^[^^28^^]^
Herbs	Votalin tablet	Pain relief	Very low	Hou, 2015^[^^14^^]^
		Function improving	Very low	Hou, 2015^[^^14^^]^
	Placebo	Pain relief	Very low	Hou, 2015^[^^14^^]^
		Function improving	Very low	Hou, 2015^[^^14^^]^
DJD (plus glucosamine)	Glucosamine	Decreasing total WOMAC scores	Very low	Zhang, 2016^[^^29^^]^
DJD (plus meloxicam and glucosamine)	Meloxicam and glucosamine	Decreasing total WOMAC scores	Very low	Zhang, 2016^[^^29^^]^
MCHF	Usual treatment	No difference in walk-related pain, WOMAC scores and Lysholm scores	Very low	Zhu, 2015^[^^30^^]^
MCHF (plus usual treatment)	Usual treatment	Pain relief	Very low	Zhu, 2015^[^^30^^]^
		Decreasing total WOMAC scores and Lequesne index	Very low	Zhu, 2015^[^^30^^]^
Chinese herbal bath	Drug therapy	Pain relief	Low	Chen, 2015^[^^31^^]^
		Higher overall effectiveness	Low	Chen, 2015^[^^31^^]^
Tai Chi	Education	Pain relief	Very low	Ye, 2014^[^^32^^]^
		Function improving	Very low	Ye, 2014^[^^32^^]^
	Usual care	Pain relief	Very low	Ye, 2014^[^^32^^]^
		Function improving	Very low	Ye, 2014^[^^32^^]^
	No treatment	Pain relief	Very low	Ye, 2014^[^^32^^]^
		Function improving	Very low	Ye, 2014^[^^32^^]^
Qigong	No treatment	Pain relief	Very low	Hou, 2015^[^^14^^]^
		Function improving	Very low	Hou, 2015^[^^14^^]^
	Sham Qigong	Pain relief	Very low	Hou, 2015^[^^14^^]^
		Function improving	Very low	Hou, 2015^[^^14^^]^

**Notes:** DJD, Duhuo Jisheng decoction; MCHF, Manufactured Chinese herbal formula.

## Discussion

### Summary of findings

Knee OA belongs to the category of Gu Bi in TCM, which refers to the pain and stiffness or malfunction of the joints. This condition mainly results from the stagnation of blood, kidney essence deficiency, and yang deficiency. In the present study, most included SRs showed that compared with sham therapy or routine therapy, TCM presents a better choice for improving symptoms with low occurrence of adverse effects. Specifically, TCM exhibited the following potential benefits for patients with knee OA: alleviating pain, improving physical function, improving quality of life, and exerting few adverse effects. However, the binary benefit outcomes, which were presupposed primary outcomes, were included in none of the included SRs or original trials.

Generally, the methodological quality has yet to be improved in certain studies. In accordance with the AMSTAR tool, two of the most obvious problems were the lack of a-priori protocol, protocol registration, as well as a list of excluded studies. By asking researchers to provide information on clinical trials regularly, a-priori protocol or protocol registration can be a potent method to enhance the transparency of trials, thus helping reduce the publication bias. The search strategy description for six SRs was also found to be unsatisfactory. Most of the SRs failed to state the date of a detailed and comprehensive search strategy for at least one database. Moreover, various sources of studies, such as grey literature, trial registers, and reference lists, were not searched in these SRs. Given these flaws, selection or reporting of bias cannot be ignored, which may raise questions regarding rigor and validity consequently. Furthermore, in accordance with the fundamental principles, TCM should be based on “syndrome differentiation”, indicating that the therapeutic methods of TCM for the same disease may vary according to the patients’ different symptoms, tongue coating, and pulse condition [33]. In our study, two included SRs only combined statistics by using the random effects model without considering the remarkable clinical heterogeneity. Nevertheless, such overall pooling of data may produce overstated or even opposite conclusions.

The risk of bias of the included SRs evaluated by ROBIS was basically corresponding to the methodological quality. However, unlike AMSTAR, ROBIS is a tool designed specifically to assess the risk of bias, and the risk of bias in the six included SRs was judged as high because they did not identify all the concerns in domains 1 to 4. For the second domain, two SRs did not report a straight and relevant study question, resulting in our doubt regarding the “comprehensive literature search.” For the third domain, the risk of bias in the two studies was high out of the inappropriate evaluation criteria. For example, the Newcastle–Ottawa Scale (NOS) used in the study of Chen et al. [31] is a quality assessment tool for non-randomized studies included in SRs [34], in which the randomization, intention-to-treat analysis, and selective reporting are not included. Hence, NOS was an inappropriate assessment tool for the included RCTs. When choosing the assessment tool, researchers need to consider whether the criteria are sufficient to identify all significant potential sources of bias. A validated tool developed specifically for trials in the SR, such as the Cochrane Back Review Group criteria, is recommended. In our study, considering that the reliability and validity of ROBIS are yet to be tested, we utilized two assessment tools to verify the findings of the methodological quality of the included SRs.

TCM might provide some benefits for patients with knee OA from the conclusions of included SRs. In addition, as discussed previously, certain TCM techniques, such as acupuncture and Tai Chi, which has been accepted in approximately one-third of elderly patients with knee OA as reported in one study [35], are explicitly listed as recommended therapies in the latest OARSI guideline. Nevertheless, the evidence levels were not satisfactory. In accordance with the GRADE approach, high or moderate quality of evidence was found in none of the 10 included SRs, mainly because of the limitations of original studies, inconsistencies among studies, imprecision in the treatment effect, and publication bias. Thus, the evidence is low or very low to support the use of TCM therapy to improve the symptoms and quality of life of patients with knee OA.

### Strength and limitations

To our knowledge, this study is the first overview that systematically reviewed SRs on TCM for patients with knee OA. We searched medical databases and hand-searched reference lists, and then summarized the findings and assessed the methodological quality, as well as the quality of evidence of the included studies by using AMSTAR, ROBIS, and GRADE approach. However, our study faced its own limitations. First, the retrieval language limited to English may generate a sampling bias. Second, our assessment relied on what SRs had reported. The authors possibly designed and conducted their SRs more completely but removed certain important details that we sought. In this case, our results might be influenced by the reporting quality of the included SRs. Third, although two reviewers in our study independently used the AMSTAR, ROBIS, and GRADE tools to assess both the quality of methodology and evidence, we should emphasize that some subjectivity may exist. However, we recorded each basis of evaluation and made frequent discussion among all authors regarding any queries to keep the process transparent.

### Implication for future research and practice

Future SRs on TCM should be well designed and conducted to support the utilization of TCM on knee OA patients. Given that TCM is a complex intervention, and knee OA is a disease treated with complex interventions, an important challenge of SRs on TCM is to deal with the potential heterogeneity among patients (mainly derived from the variation of the state of the syndrome), interventions (such as the acupoint selection, methods of delivery, and qualification of implementers), and outcomes (validated scales or defined by the authors). When conducting SRs, authors should analyze the source of heterogeneity initially; thus, potentially effective approaches, including subgroup analysis, meta-regression, or descriptive analysis, can be used to explore this complexity. Moreover, none of the included SRs reported the binary benefit outcomes. In this case, the efficacy of TCM for the treatment of knee OA cannot be adequately evaluated. We strongly recommend that in future studies, including both SRs and primary trials, authors should consider these core outcomes for further assessment. Additionally, information on the adverse events provided was limited, which might resulted from the lack of detailed information in primary trials. The lack of guidelines on reporting these events in SRs should also be noticed. Thus far, the Preferred Reporting Items for Systematic Review and Meta-analyses (PRISMA), a reporting guideline specific for SRs, has mainly focused on efficacy and not on harms [36]. Hence, to get a balanced evaluation of an intervention, we emphasize here the crucial importance of the development of a standardized format for reporting adverse events in SRs. Finally, the methodology and quality of evidence from other SRs on TCM [37–39] were likewise problematic, which may suggest the deficiency of education or training for TCM researchers who conduct SRs and original clinical trials. We strongly recommend that relevant training should be provided to develop the TCM researchers’ consciousness and abilities in designing, conducting, and reporting TCM studies.

## Conclusions

In summary, published SRs described the potential benefits of TCM for patients with knee OA as follows: pain relief, functional improvement, and presence of few adverse events. However, the evidence is not robust enough because of the methodological flaws in primary clinical trials and SRs. Hence, these conclusions on available SRs should be treated with caution for clinical practice. Furthermore, future clinical trials and SRs should be rigorously and prudently designed and conducted.

## Supporting information

S1 Table Characteristics of original RCTs. (DOC)

S2 Table Evidence profile: Quality of evidence in included SRs assessed by GRADE. (DOC)

S3 Table PRISMA checklist. (DOC)
