# Supplementary material for: Traditional Chinese medicine for knee osteoarthritis: An overview of systematic review
Source: PLoS One. 2017 Dec 21;12(12):e0189884. doi: 10.1371/journal.pone.0189884 (PMC5739454; doi:10.1371/journal.pone.0189884)
Supplement: S1 Table — (DOC) [file pone.0189884.s001.doc]

**S1 Table. Characteristics of original RCTs**

**I.** **Characteristics of original RCTs for general TCM**

| **SRs** | **Original RCTs** | **Number of patients (T1/T2/C)** | **Age**  **(yrs)**  **(E1/E2/C)** | **Gender**  **(M/F)** | **Interventions** | | **Primary outcomes** |
| --- | --- | --- | --- | --- | --- | --- | --- |
|  |  |  |  |  | **Treatment group** | **Control group** |  |
| Hou,  2015^[14]^ | Ng  2003 | 24(8/8/8) | 75-96 | 1/23 | EA;  EA TENS  (ST-35 and EX-LE-4) | General education on osteoarthritic knee care | Pain: numerical rating scale of pain Function: passive range of movement of the OA knee; the Timed Up-and-Go test (TUGT) |
|  | Berman  2004 | 570(190/191/189) | ≧50 | M: 40% | Acupuncture  (5 local and 4 distant points) | Sham acupuncture;  Education | Pain: WOMAC pain score  Function: WOMAC function score; SF-36; the 6-min walk time; the patient global assessment |
|  | Tukmachi  2004 | 29(9/10/10) | ≧18 | F: 82.8% | Acupuncture alone;  acupuncture and their existing analgesic and anti-inflammatory medication | (1) Week 1-5: current medications,  (2)Week 6-10: acupuncture plus  medications | Pain: VAS(10-cm)  Function: WOMAC; global assessment |
|  | Vas  2004 | 97(48/49) | ≧45 | F: 83.5% | Acupuncture plus diclofenac | Placebo acupuncture plus diclofenac | Pain: VAS(100-mm)  Function: WOMAC stiffness and function scale  Others: the dosage of diclofenac accumulated; the profile of quality of life in the chronically ill (PQLC) |
|  | Witt  2005 | 300(150/76/74) | 50-75 | F: 66.3% | Acupuncture  Minimal acupuncture | Waiting list | Pain and function: WOMAC; Pain Disability Index (PDI); SF-36  Others: Schmerzempfindungs -Skala [SES]; Allgemeine; Depressionsskala [ADS]; questions about workdays lost |
|  | Scharf  2006 | 1039(342/330/367) | ≧40 | F: 68.8% | TCA ＋ conservative therapy | Conservative therapy;  Sham acupuncture＋ conservative therapy | Pain: WOMAC pain scale  Function: WOMAC function scale  Others: the dichotomized global patient assessment; SF-12 physical subscale |
|  | Williamson  2007 | 181(60/60/61) | na | F: 53.6% | Acupuncture | Physiotherapy;  Standard management (exercise and advice by consensus) | Pain: VAS(10-cm) Function:Oxford Knee Score (OKS); WOMAC; a 50-m timed walk  Others:Hospital Anxiety and Depression score (HAD) |
|  | Jubb  2008 | 44(34/10) | ≧18 | na | Acupuncture  (distal and local acupunctureoints) | Sham acupuncture | Pain: VAS Function: WOMAC; EuroQol Others: plasma ß-endorphin concentration |
|  | Itoh  2008 | 30(10/10/10) | >60 | na | Standard acupuncture;  Trigger point acupuncture | Sham acupuncture | Pain: VAS(100-mm)  Function: WOMAC |
|  | Lu  2010 | 20(10/10) | T: Mean 63.4 (SD 5.6)  C: Mean 64.3  (SD 6.3) | na | EA | Sham EA | Pain: VAS(10-cm)  Function: Gait analysis (Vicon 512,  Oxford Metrics, UK) |
|  | Soni  2012 | 56(28/28) | na | na | Acupuncture and exercise therapy | Standard exercise and advice leaflet | Pain: VAS(10-cm)  Function: Oxford Knee Score (OKS); 50 m timed walk  Others: the Hospital Anxiety and Depressio score (HAD) |
|  | Mavrommatis  2012 | 120(30/30/30) | Mean 62.3  (SD 9.9) | 12/27 | Acupuncture and etoricoxib | Sham acupuncture and etoricoxib;  Etoricoxib only | Function: WOMAC stifness and function scale; Short Form-36 version 2  Others: BMI, CBC, ESR, Rh factor, liver and kidney function, uric acid and arterial blood pressure |
|  | Wang  2012 | 150(60/60/30) | 59.3 | F: 91% | FNZG;  SJG | Drug therapy (Oral diclofenac sodium) | Pain: VAS(100-mm)  Function: WOMAC  Others: TCM Syndrome Questionnaire |
|  | Park  2009 | 57(31/26) | 59.90/58.92 | F: 90.32% / 90.31% | AIF | Placebo | Pain: VAS(100-mm)  Function: K-WOMAC |
|  | Li  2010 | 100(50/50) | T: Mean 52.46  (SD 9.86)  C: Mean 53.16  (SD 10.05) | 47/53 | Chinese medicine  (orally taken) | Votalin tablet | Pain (0-3)  Morning stiffness (0-3)  Walking (0-3)  Stand up from sitting position (0-3)  Walk unrestrictedly (0-3)  Lassitude in the loin and knees (0-3)  Daily activity (0-3)  Carry out all kinds of activities (0-3) |
|  | Chen  2008 | 112(60/52) | 63.9/58.8/62.9 | 30/76 | Qigong | A sham healer to mimic qigong | Pain: McGill Pain Questionnaire (MPQ-SF)  Function: WOMAC; Time to walk a 15-m straight path; ROM when squatting down to the lowest position without pain  Others: the Spielberger State Trait; Anxiety Scale; the Center for Epidemiologic Studies Depression Scale; an adopted general mood index |
|  | An  2008 | 28(14/14) | T: Mean 65.4  (SD 8.2)  C: Mean 64.6  (SD 6.7) | na | Baduanjin | No treatment | Pain: WOMAC pain scale  Function: WOMAC; SF-36; 6-MWT; ISKE |
|  | Lee  2009 | 41(28/13) | 69.1±5.4 | F: 93% | Tai Chi | No treatment | Pain: No pain score.  Function: WOMAC; The 6-m walking test  Others: Short Form 36 (SF-36); Health Surve |

Footnotes: EA: electro-acupuncture; VAS: visual analogue scale; WOMAC: The Western Ontario and McMaster Universities Arthritis Index; ACR: American College of Rheumatology, K-L: Kellgren-Lawrence criteria.

**Ⅱ. Characteristics of original RCTs for acupuncture**

| **SRs** | **Original RCTs** | **Number of patients (T1/T2/C1/C2)** | **Age (yrs)**  **(T/C)** | **Gender**  **(M/F)** | **Interventions** | | **Primary outcomes** |
| --- | --- | --- | --- | --- | --- | --- | --- |
|  |  |  |  |  | **Treatment group** | **Control group** |  |
| Cao,  2012^[24]^ | Berman 1999 | 73(36/37) | 65.0 | F: 60.3% | MA, EA | Waiting list | WOMAC |
|  | Berman 2004 | 570(190/191/189) | 65.5 | F: 64.0% | MA, EA | Sham acupuncture;  Usual care | WOMAC |
|  | Foster 2007 | 352(117/119/116) | 63.5 | F: 61.4% | MA | Sham acupuncture;  Usual care | WOMAC |
|  | Jubb 2008 | 68(34/34) | 65.1 | F: 70.5% | MA, EA | Sham acupuncture; | WOMAC |
|  | Lansdown 2009 | 30(15/15) | 63.5 | F: 60.0% | MA | Usual care | WOMAC |
|  | Sangdee 2002 | 193(49/48/49/47) | 63.0 | F: 77.7% | EA | Sham acupuncture*;  Sham acupuncture† | WOMAC |
|  | Scharf 2006 | 1039(330/367/342) | 63.0 | F: 68.8% | MA | Sham acupuncture;  Usual care | WOMAC |
|  | Suarez-Almazor 2010 | 527(153/302/72) | 64.5 | F: 64.1% | EA | Sham acupuncture;  Waiting list | WOMAC |
|  | Takeda 1994 | 40(20/20) | 62.0 | F: 50.0% | MA | Sham acupuncture | WOMAC |
|  | Tukmachi 2004 | 29(19/10) | 62.0 | F: 82.8% | MA | Waiting list | WOMAC |
|  | Vas 2004 | 97(48/49) | 67.0 | F: 83.5% | MA, EA | Sham acupuncture | WOMAC |
|  | Williamson 2007 | 181(60/60/61) | 71.0 | F: 53.6% | MA | Usual care‡;  Usual care§ | WOMAC |
|  | Witt 2005 | 294(149/75/70) | 64.0 | F: 66.3% | MA | Sham acupuncture;  Waiting list | WOMAC |
|  | Witt 2006 | 342(175/167) | 61.0 | F: 60.5% | MA | Waiting list | WOMAC |
| Manheimer,  2007^[25]^ | Berman 1999 | 36 | 65 | M: 40% | Formula with EA | Waiting list | WOMAC |
|  | Berman 2004 | 190 | 65 | M: 36% | Formula with EA | Sham acupuncture;  Usual care | WOMAC |
|  | Christensen 1992 | 17 | 69 | M: 31% | Formula acupuncture | Sham acupuncture;  Usual care | WOMAC |
|  | Molsberger 1994 | 71 | 60 | M: 37% | Formula acupuncture | Sham acupuncture;  Usual care | WOMAC |
|  | Sangdee 2002 | 97(49/48) | 63 | M: 22% | Formula with EA | Sham acupuncture*;  Sham acupuncture† | WOMAC |
|  | Scharf 2006 | 330 | 63 | M: 31% | Flexible formula | Sham acupuncture;  Usual care | WOMAC |
|  | Takeda 1994 | 21 | 62 | M: 50% | Formula | Sham acupuncture | WOMAC |
|  | Tukmachi 2004 | 20(10/10) | 62 | M: 17% | Formula | Waiting list | WOMAC |
|  | Vas 2004 | 48 | 67 | M: 16% | Formula with EA | Sham acupuncture | WOMAC |
|  | Witt  2005 | 150 | 64 | M: 34% | Flexible formula | Sham acupuncture;  Waiting list | WOMAC |
|  | Witt  2006 | 175 | 61 | M: 40% | Individualized | Waiting list | WOMAC |
| Yamashita,  2006^[26]^ | Christensen 1992 | 32 | 69 | M: 31% | No treatment | Sham acupuncture  Usual care | Adverse events |
|  | Sangdee 2002 | 91(46/45) | 63 | M: 22% | Dummy electroacupuncture | Sham acupuncture*;  Sham acupuncture† | Adverse events |
|  | Tukmachi 2004 | 19(9/10) | 62 | M: 17% | acupuncture and medication | Waiting list | Adverse events |
|  | Vas 2004 | 97(48/49) | 67 | M: 16% | Streitberger needle | Sham acupuncture | Adverse events |
|  | Berman 2004 | 379(190/189) | 65 | M: 36% | sham acupuncture | Sham acupuncture;  Usual care | Adverse events |
|  | Witt 2005 | 224(150/74) | 64 | M: 34% | minimal acupuncture | Sham acupuncture;  Waiting list | Adverse events |
|  | Scharf 2006 | 691(326/365) | 63 | M: 31% | minimal acupuncture | Sham acupuncture;  Usual care | Adverse events |

Footnotes: MA: manual acupuncture; EA: electro-acupuncture; *sham acupuncture with co-intervention of diclofenac; †sham acupuncture with co-intervention of placebo diclofenac; ‡physiotherapy, §advice and exercise

**Ⅲ. Characteristics of original RCTs for moxibustion**

| **SRs** | **Original RCTs** | **Number of patients (T/C)** | **Age**  **(yrs)** | **Gender**  **(M/F)** | **Interventions** | | **Primary outcomes** |
| --- | --- | --- | --- | --- | --- | --- | --- |
|  |  |  |  |  | **Treatment group** | **Control group** |  |
| Song,  2016^[27]^ | Chen 2015a | 288(144/144) | T:Mean 55.0  (SD 5.1)  C:Mean 56.0  (SD 5.0) | T: 88/56  C: 87/57 | Heat-sensitive moxibustion  (SP9, GB34, ST34, SP10) | Drug therapy (Intra-articular injection of sodium hyaluronate) | Response rate-GPCRND  Knee circumference  AEs |
|  | Chen 2015b | 288(144/144) | T:Mean 53.0  (SD 5.2)  C:Mean 56.0  (SD 5.0) | T: 91/53  C: 87/57 | Conventional moxibustion  (EX-LE5, EX-LE2) | Drug therapy (Intra-articular injection of sodium hyaluronate) | Response rate-GPCRND  Knee circumference  AEs |
|  | Kim 2014 | 212(102/110) | T:median 56  (52–62)  C:median 57  (51–62) | T: 17/85  C: 16/94 | Conventional moxibustion  (ST35, ST34, SP9, ExLE04, SP10, Ashi point) | Conventional care | WOMAC Physical function Pain-NRS |
|  | Ren 2010 | 100(50/50) | 41-75 | na | Conventional moxibustion  (EX-LE4, EX-LE5,ST34, SP10, ST35, SP9, GB34; 4 points were adopted at every treatment) | Drug therapy (Oral diclofenac sodium) | QoL  AEs  Response rate |
|  | Ren 2015 | 136(69/67) | T:Mean 65.6  (SD 7.4)  C:Mean 64.1  (SD 8.7) | T: 20/49  C: 23/44 | Conventional moxibustion  (ST 35, EX-LE4, Ashi point) | Sham moxibustion | QoL  AEs |
|  | Sun 2008 | 56(29/27) | T:Mean 59.9  (SD 8.6)  C:Mean 61.7  (SD 9.1) | T: 11/18  C: 12/15 | Herbal cake-separated moxibustion (EX-LE4,  ST35, SP9, GB34, SP10,  ST34, EX-LE2, BL18,  BL23; 2–4 points were  adopted at every treatment) | Drug therapy (Oral diclofenac sodium) | Response rate |
|  | Wu 2011 | 50(24/26) | T:Mean 47.3  (SD 9.6)  C:Mean 46.9  (SD 11.6) | T: 10/14  C: 12/14 | Heat-sensitive moxibustion  (EX-LE4,  ST35, ST34, SP9, GB34,  SP10, Ashi point) | Drug therapy (Intra-articular injection of sodium hyaluronate) | Response rate  Pain |
|  | Zhang 2011 | 60(30/30) | 46-73 | T: 22/38 | Conventional moxibustion | Drug therapy  (Oral celecoxib) | Response rate  Pain-VAS |
|  | Zhao 2014 | 110(55/55) | T:Mean 65.8  (SD 7.5)  C:Mean 64.6  (SD 8.4) | T: 16/39  C: 21/34 | Conventional moxibustion | Sham moxibustion | AEs  Pain-WOMAC  Physical function-WOMAC |
|  | Zhou 2010 | 70(35/35) | T:Mean 59.0  (SD 10.0)  C:Mean 61.0  (SD 9.0) | T: 15/20  C: 12/23 | Herbal cake-separated  Moxibustion  (ST 35, EXLE4, Ashi point) | Drug therapy (Oral diclofenac sodium) | AEs  Response rate  Pain-NRS  Knee circumference |
|  | Zhou 2014 | 61(39/22) | T:Mean 67.0  (SD 10.0)  C:Mean 66.0  (SD 12.0) | T: 14/25  C: 5/17 | Conventional moxibustion  ( RN8, ExLE4, ST35, SP10, ST34) | Drug therapy  (Oral celecoxib) | Pain-VAS  Physical function-CDTEDS |
|  | Cheng 2008 | 120(60/60) | T:Mean 57.8  (SD 5.4)  C:Mean 58.8  (SD 5.7) | T: 11/49  C: 16/44 | Sandwiched moxibustion  ( EX-LE4,  EX-LE5, Ex-LE2, SP9,  GB34) | Drug therapy (Oral diclofenac sodium) | Response rate  Pain-NRS |
|  | Yang 2008 | 64(33/31) | T:Mean 59.3  (SD 9.5)  C:Mean 59.3  (SD 9.0) | T: 14/19  C: 11/20 | Herbal cake-separated  Moxibustion  ( EX-LE5,  Ex-LE2, SP9, GB34, SP10,  ST36; 2–4 points were  adopted at every treatment) | Drug therapy (Oral diclofenac sodium) | Response rate |
| Li,  2016^[28]^ | Zhao  2014 | 110(55/55) | T:Mean 65.8  (SD 7.5)  C:Mean 64.6  (SD 8.4) | T: 16/39  C: 21/34 | Conventional moxibustion | Sham moxibustion | WOMAC scale |
|  | Ren  2015 | 136(69/67) | T:Mean 65.6  (SD 7.4)  C:Mean 64.1  (SD 8.7) | T: 20/49  C: 23/44 | Conventional moxibustion  (ST 35, EX-LE4, Ashi point) | Sham moxibustion | SF-36 scale |
|  | Kim  2014 | 212(102/110) | T:median 56  (52–62)  C:median 57  (51–62) | T: 17/85  C: 16/94 | Conventional moxibustion  (ST35, ST34, SP9, ExLE04, SP10, Ashi point) | Conventional care | K-WOMAC scale Physical function Pain-NRS BDI  Test SF-36v2 scale |
|  | Chen  2015 | 288(144/144) | T:Mean 53.0  (SD 5.2)  C:Mean 56.0  (SD 5.0) | T: 91/53  C: 87/57 | Conventional moxibustion  (EX-LE5, EX-LE2) | Drug therapy (Intra-articular injection of sodium hyaluronate) | GPCRND-KOA scale  Knee circumference |

Footnotes: na: not available; ACR: American College of Rheumatology; GPCRND: guiding principles of clinical research on new drugs; NRS: numerical rating scale; QoL: quality of life; VAS: visual analog scale; WOMAC: Western Ontario and McMaster Universities Questionnaire; KOA: knee osteoarthritis

**IV. Characteristics of original RCTs for Chinese herbal medicine**

| **SRs** | **Original RCTs** | **Number of patients (T1/T2/C1/C2)** | **Age** (yrs)  (T/C) | **Gender**  **(M/F)** | **Interventions** | | **Primary outcomes** |
| --- | --- | --- | --- | --- | --- | --- | --- |
|  |  |  |  |  | **Treatment group** | **Control group** |  |
| Zhang,  2016^[29]^ | Teekachunh-atean  2004 | 200(100/100) | 62.66/  62.38 | T: 22/78  C: 19/81 | Duhuo Jisheng wan | Diclofenac 25 mg/time | Lequesne  VAS  AE |
|  | Yu  2010 | 113(56/57) | 56/59 | T: 21/35  C: 19/38 | Duhuo Jisheng decoction | Glucosamine 0.5 g/time | Lequesne ADR |
|  | Cao  2013 | 100(50/50) | 61.5/  57.38 | T: 24/26  C: 23/27 | Duhuo Jisheng decoction ＋ Sodium hyaluronate | Sodium hyaluronate 2 mL/time | VAS ADR |
|  | Gu  2013 | 60(30/30) | 57.38/  54.98 | T: 13/17  C: 12/18 | Duhuo Jisheng decoction | Meloxicam | Lysholm VAS |
|  | Yu  2013 | 43(21/22) | 55.2/57.2 | T: 8/13  C: 10/12 | Duhuo Jisheng decoction ＋ Glucosamine | Glucosamine 2 pills/time | WOMAC |
|  | Zhang  2013 | 80 (40/40) | 57/56 | T: 11/29  C: 12/28 | Duhuo Jisheng decoction ＋ Diacerein | Diacerein 50 mg/time | WOMAC VAS ADR |
|  | Zhong  2013 | 56 (28/28) | 70/71 | T: 10/18  C: 11/17 | Duhuo Jisheng decoction ＋ Glucosamine | Glucosamine 2 pills/time | WOMAC |
|  | Dong  2014 | 60 (30/30) | 55.1 /51.26 | T: 9/21  C: 8/22 | Duhuo Jisheng decoction ＋ Knee arthroscopic surgery + rehabilitation training | Knee arthroscopic surgery + rehabilitation training | Lysholm |
|  | Huang  2012 | 70 (35/35) | 56.4/  53.26 | T: 12/23  C: 15/20 | Duhuo Jisheng decoction ＋ Sodium hyaluronate | Sodium hyaluronate 20 mg/time | Lysholm VAS |
|  | Jiang  2014 | 40 (20/20) | 63.15/  62.3 | T: 3/17  C: 3/17 | Duhuo Jisheng decoction ＋ Meloxicam | Meloxicam 7.5 mg/time | WOMAC  ADR |
|  | Wang  2014 | 100 (50/50) | 65.12/  64.3 | T: 21/29  C: 24/26 | Duhuo Jisheng decoction ＋ Glucosamine | Glucosamine 2 pills/time | WOMAC |
|  | Zhou  2014 | 60 (30/30) | na | na | Duhuo Jisheng decoction ＋ Meloxicam | Meloxicam 15 mg/time | WOMAC  ADR |
| Zhu,  2015^[30]^ | Zuo  2015 | 46/47 | T: Mean 56.22 (SD 9.92)  C: Mean 58.58 (SD 8.84) | T: 7/34  C: 6/32 | Fufang Xiatianwu pill (0.6 g, tid, 12 W) | Diclofenac sodium (25 mg, tid, 12 w) | VAS-2  SF-36  WOMAC |
|  | Huang  2015 | 34/34 | T: 53-76 (68) C: 53-76 (65) | T: 19/15 C: 18/16 | Huo xue zhi tong capsule (2 pills, tid, 4 w) + CI | Sodium hyaluronate  (IA, 2 mL, qw, 4 w) | VAS-1 |
|  | Cao  2015 | 30/30/30 | T1: Mean 61.57 (SD 6.68)  T2: Mean 61.03 (SD 5.89)  C: Mean 61.41 (SD 7.15) | T1: 8/22  T2: 9/21  C: 6/24 | T1: Jin tian ge capsule (3 pills, tid, 1 m);  T2: T1 + CI | Aceclofena (0.1 g, bid, 1 m) | Lequesne |
|  | Zhang  2013 | 63/61 | T: 38–70 (56.3)  C: 39–73 (57.6) | T: 13/50  C: 12/49 | Pan long qi pill (0.9 g, tid, 4 w) + CI | Diacerein (50 mg, bid, 4 w) + Aceclofenac(0.1 g, bid, 4 w) | WOMAC |
|  | Yi  2013 | 30/30 | T: 40–70  (54.5)  C: 39–72  (55.3) | T: 9/21  C: 8/24 | Pan long qi pill (3 pills, tid, 6 w) | Glucosamine (250 mg, tid, 6 w) | VAS-1  VAS-2 |
|  | Niu  2013 | 27/33 | na | na | Jin gu tong xiao pill (6 g, bid, 1 m) + CI | Glucosamin (2 pills, tid, 1 m) | Lequesne  SF-36 |
|  | Liao  2013 | 230/230 | T: Mean 57.67 (SD 7.62)  C: Mean 57.43 (SD 7.81) | T: 105/125  C: 101/129 | Jiangu granule (12 g, tid, 1 m) | Sodium hyaluronate (IA, 20 mg, qw, 1 m) | VAS-1 |
|  | Li  2013 | 105/105 | T: 38–54 (45.0)  CG: 40–52 (46.3) | T: 70/35  C: 72/33 | Hua mo yan granule (12 g, tid, 4 w) | Ketoprofen (50 mg, tid, 4 w) | VAS-1 |
|  | Li  2013 | 93/93 | T: Mean 56.2  (SD 6.7)  C: Mean 54.8  (SD 7.2) | T: 40/53  C: 38/55 | Hua mo yan granule (12 g, tid, 4 w) + CI | Infrared therapy (15 min, bid) | VAS-1 |
|  | Li  2013 | 50/50/50/50 | T1: Mean 64.23 (SD 1.69)  T2: Mean 64.56 (SD 1.75)  C1: Mean 64.35 (SD 1.78)  C2: Mean 63.85  (SD 1.72) | T1: 22/28  T2: 23/27  C1: 23/27  C2: 24/26 | T1: Fu gui gu tong granule (5 g, tid, 1 m)  T2: T1 + C2 | C1: Diclofenac sodium (25 mg, tid, 1 m)  C2: Ozone (IA, 10 mL, qw, 1 m) | VAS-1  WOMAC |
|  | Chen  2013 | 48/48/48 | T1: Mean 58 (SD 17.7)  T2: Mean 61 (SD 18.6)  C: Mean 61 (SD 16.0) | T1: 17/25  T2: 15/25  C: 14/28 | T1: Xian ling gu bao capsule (1.5 g, bid,  4 w)  T2: T1 + CI | Ozone (IA, 15 mL, qw, 4 w) | VAS-1  Lysholm |
|  | Luo  2012 | 33/34/36 | T1: 41–74 (51.93)  T2: 42–67 (50.15)  C: 39–65 (50.35) | T1: 15/18  T2: 17/17  C: 17/19 | T1: Xian ling gu bao capsule (1.5 g, bid)  T2: T1 + CI | Sodium hyaluronate (IA, qw, 5 w) | VAS-1  VAS-2  WOMAC |
|  | Lu  2012 | 120/120 | T: Mean 52.93  (SD 14.22)  C: Mean 54.01 (SD 15.35) | T: 55/65 C: 50/70 | Teng huang jian gu pill (1.5–3.0 g, bid,  4 w) | Celecoxib (200 mg, qd, 4 w) | WOMAC |
|  | Li  2012 | 58/31 | T: Mean 59.4  (SD 14.8)  C: Mean 63.2 (SD 10.3) | T: 22/36 C: 11/2 | Fufang Xiao Huo Luo pill (2 pills, bid, 2 pm) | Glucosamin (250 mg, bid, 2 m) | VAS-1  VAS-2  Lyshom |
|  | Li  2012 | 75/77 | T: 46–73 (55.2)  C: 43–72 (54.1) | T: 22/53 C: 23/54 | Hua mo yan granule (12 g, tid, 4 w) + CI | Infrared therapy (15 min, bid, 4 w) | VAS-1 |
|  | Jiang  2012 | 55/55 | T: Mean 61.92  (SD 9.89)  C: Mean 62.78  (SD 10.25) | T: 33/22 C: 31/24 | Bai shao zong gan capsule (0.3 g, tid,  4 w) + CI | Celecoxib (200 mg, qd, 4 w) | VAS-1 |
|  | Wang  2011 | 49/49 | Mean 56.9 (SD 13.0) | 48/50 | Jin tian ge capsule (3 pills, tid, 3 m) + CI | Alfacalcidol (0.5 µg, qd) + Naproxen (0.25, bid) | Lequesne |
|  | Kang  2011 | 72/46/42 | T1: Mean 54.34 (SD 6.93)  T2: Mean 52.63 (SD 7.29)  C: Mean 54.24 (SD 6.80) | T1: 14/58  T2: 10/32  C: 12/34 | T1: Wang bi pill (2.0 g, tid, 8 w)  T2: T1 + CI | Diclofenac sodium (25 mg, qd/bid, 8 w) | VAS-1 |
|  | Yan  2010 | 30/30 | T: Mean 57.90 (SD 7.10)  C: Mean 59.87 (SD 7.24) | T: 6/23  C: 6/24 | Kang gu zeng sheng capsule (5 pills, tid,  4 w) | Glucosamine (2 pills, tid, 4 w) | WOMAC |
|  | Xiao  2009 | 77/77 | T: Mean 56.20  (SD 10.64)  C: Mean 57.49 (SD 12.82) | T: 21/53 C: 18/52 | Pan long qi pill (3 pills, tid, 6 w) | Diclofenac sodium (25 mg, tid, 6 w) | VAS-2 |
|  | Qi  2009 | 60/60 | T: 45–69  C: 46–70 | T: 13/47 C: 11/4 | Xin huang pill (1.28 g, tid, 15 d) | Ibuprofen (0.6 g, bid, 15 d) + Indometacin (25 mg, tid, 15 d) | Lysholm |
|  | Li  2009 | 50/50 | T: 45–68 (51.8)  C: 46–67 (52.6) | T: 22/28  C: 23/27 | Bi qi capsule (1.2 g, tid, 6 w) | Glucosamine (250 mg, tid, 6 w) | HSS |
|  | Liu  2007 | 66/64 | T: Mean 59.5  (SD 9.4)  C: Mean 58 (SD 8.5) | T: 23/43 C: 26/38 | Jin gu tong xiao pill (6 g, bid, 2 m) + CI | Sodium hyaluronate (IA, 5 mL, q4w, 4 w) | Lysholm |
|  | Chen  2007 | 37/34 | 47–76 | 24/47 | Gu jin pill (0.9 g, tid, 8 w) | Diclofenac sodium (75 mg, qd, 8 w) | Lysholm |
|  | Yang  2006 | 60/60 | T: 45–68 (51.2)  C: 46–65  (53.1) | T: 28/32  C: 26/34 | Pan long qi pill (3 pills, tid, 6 w) | Glucosamine (250 mg, tid, 6 w) | VAS-1  VAS-2 |
|  | Xu  2004 | 30/30 | 50-79 | 25/35 | Bai shao zong gan capsule (0.6 g, bid,  6 w) | Nabumetone (1.0 g, qd, 6 w) | VAS-1 |

Footnotes: na: not available; ACR: American College of Rheumatology; GPCRND: guiding principles of clinical research on new drugs; NRS: numerical rating scale; QoL: quality of life; VAS: visual analog scale; WOMAC: Western Ontario and McMaster Universities Questionnaire; KOA: knee osteoarthritis; ADR, adverse drug reaction; AE, adverse event; Bid, twice a day; Qd, once a day; T, treatment group; Tid, three times a day

**Ⅴ. Characteristics of original RCTs for** **Chinese herbal bath therapy**

| **SRs** | **Original RCTs** | **Number of patients (T/C)** | **Age**  **(yrs)** | **Gender**  **(M/F)** | **Interventions** | | **Primary outcomes** |
| --- | --- | --- | --- | --- | --- | --- | --- |
|  |  |  |  |  | **Treatment group** | **Control group** |  |
| Chen,  2015^[31]^ | Liang  2010 | 60(30/30) | 60 | na | 8 herbs: 20–50 g each  Steamed and bathed for  30 min | Salicylic acid glycol patch | (1) VAS pain  (2) Lysholm’s score  (3) Traditional Chinese medicine assessment |
|  | Xiong  2010 | 120(60/60) | 61 | F: 78% | 17 herbs: 20 g each  Steamed and bathed | Diclofenac sodium 75 mg | Traditional Chinese medicine assessment |
|  | Wang  2010 | 55(30/25) | 60 | F: 69% | 8 herbs: 15 g each  Steamed and bathed for 30 min | Hyaluronate injection | Traditional Chinese medicine assessment |
|  | He  2011 | 90(45/45) | 59 | F: 47% | 12 herbs: 6–20 g each  Steamed and bathed for  30 min | Diclofenac diethylamine gel | (1) Lysholm’s score  (2) Traditional Chinese medicine assessment |
|  | Huang  2011 | 120(60/60) | 58 | F: 57% | 31 herbs: 9–18 g each  Steamed and bathed for  20 min | Hyaluronate injection Combined with  triamcinolone injection | Japanese orthopedic association assessment |
|  | Li  2011 | 204 | 63 | F: 60% | 14 herbs: 9–30 g each  Steamed and bathed for  30 min | Loxoprofen, 60 mg | VAS pain |
|  | Wu  2011 | 98(55/43) | 55 | F: 51% | 8 herbs: 20 g each  Steamed and bathed for  40 min | Meloxicam 7.5 mg | Traditional Chinese medicine assessment |
|  | Zhang  2011 | 90(45/45) | 58 | F: 48% | 8 herbs: 5–15 g each  Steamed and bathed | Diclofenac sodium 75 mg | Lysholm’s score |
|  | Chen  2012 | 120(60/60) | 56 | F: 53% | 12 herbs: 10–30 g each  Steamed and bathed for  30 min | Diclofenac diethylamine gel | Traditional Chinese medicine assessment |
|  | Wang  2012 | 73(43/30) | 62 | F: 49% | 12 herbs: 10–30 g each  Steamed and bathed for  30 min | Nimesulide 100 mg | Japanese orthopedic association assessment |
|  | Bai  2013 | 132(67/65) | 53 | 78% | 19 herbs: 10–30 g each  Steamed and bathed for  30 min | Diclofenac diethylamine gel | Traditional Chinese medicine assessment |
|  | Liao  2013 | 96(48/48) | 57 | 59% | 17 herbs: 3–30 g each  Steamed and bathed for  30 min | Diclofenac sodium 25 mg | (1) VAS pain  (2)Traditional Chinese medicine assessment |
|  | Wei  2013 | 90 | 62 | 73% | 7 herbs: 10–20 g each  Steamed and bathed for  30 min | Ibuprofen 0.3 g | Traditional Chinese medicine assessment |
|  | Wang  2013 | 100 | 63 | 84% | 16 herbs: 10–30 g each  Steamed and bathed for  40 min in treatment  machine | Glucosamine Hydrochloride 480 mg | VAS pain |
|  | Xie  2014 | 200(100/100) | 59 | 67% | 13 herbs: 10–15 g each  Steamed and bathed for  20 min in treatment  machine | Meloxicam 7.5 mg | Lysholm’s score |

Footnotes: na: not available; VAS: visual analog scale; WOMAC: Western Ontario and McMaster Universities Questionnaire

**Ⅵ. Characteristics of original RCTs for Tai Chi**

| **SRs** | **Original RCTs** | **Number of patients (T/C)** | **Age (yrs)**  **(T/C)** | **Gender**  **(M/F)** | **Interventions** | | **Primary outcomes** |
| --- | --- | --- | --- | --- | --- | --- | --- |
|  |  |  |  |  | **Treatment group** | **Control group** |  |
| Ye,  2014^[32]^ | Ni  2010 | 35(18/17) | 62.89/63.47 | F: 100% | 24-form simplified Yang style TC | Attention controlled | Physical function WOMAC total WOMAC pain WOMAC stiffness WOMAC function |
|  | Wang  2009 | 35(20/15) | 63/68 | M/F: 1/3 | 10 forms from Yang style TC | Wellness education & stretching | Stair climb time 6-min walk distance WOMAC Pain Physical function Patient VAS Physician VAS Chair stand time CES-D SF 36-PCS |
|  | Brismee  2007 | 41(22/19) | 70.8/68.8 | 86.4% female (T) 78.9% female (C) | 24-form simplified Yang style TC | Health related lecture | Pain (VAS) Maximum Minimum Overall Physical function (WOMAC) Pain Stiffness Physical function Overall Knee ROM |
|  | Song  2010 | 82(41/41) | 63.03/61.20 | F: 100% | 31 forms of Sun-style TC with qigong breathing exercise | Self-help education | Knee muscle strength extensor flexor Knee muscle endurance Ward’s triangle trochanter |
|  | Lee  2009 | 44(29/15) | 70.2/66.9 | M/F:  93.1% (T) 93.3% (C) | 18 TC movements incorporating intention of movement, enhancing awareness of breathing | Waiting list | Quality of life SF-36 Physical function WOMAC pain Physical performance 6-min walk test |
|  | Song  2003 | 43(22/21) | 64.8/62.5 | F: 60% | 12 movements in Sun style TC | Routine treatment | K-WOMAC pain stiffness physical Physiological measures Balance Abdominal muscle strength |

Footnotes: na: not available; TC: Tai Chi; VAS: visual analog scale; WOMAC: Western Ontario and McMaster Universities Questionnaire
