# Supplementary material for: Traditional Chinese medicine for knee osteoarthritis: An overview of systematic review
Source: PLoS One. 2017 Dec 21;12(12):e0189884. doi: 10.1371/journal.pone.0189884 (PMC5739454; doi:10.1371/journal.pone.0189884)
Supplement: S2 Table — (DOC) [file pone.0189884.s002.doc]

**S2 Table. Evidence profile: quality of evidence in included SRs assessed by GRADE**

| Intervention | Control | Main findings | Risk of bias | Inconsistency | Indirectness | Imprecision | Publication bias | Quality of evidence | Study ID |
| --- | --- | --- | --- | --- | --- | --- | --- | --- | --- |
| Acupuncture | sham acupuncture; education; physiotherapy; exercise; usual care | Pain relieving | Serious^1^ | Not serious | Not serious | Not serious | Serious^2^ | Low | Hou, 2015^[14]^ |
| Acupuncture | sham acupuncture; education; physiotherapy; exercise; usual care | Function improving | Serious^3^ | Serious^4^ | Not serious | Not serious | Serious^2^ | Very low | Hou, 2015^[14]^ |
| Herbs | Placebo; Votalin tablet | Pain relieving | Serious^5^ | Serious^4^ | Not serious | Serious^6^ | Serious^2^ | Very low | Hou, 2015^[14]^ |
| Herbs | Placebo; Votalin tablet | Function improving | Serious^7^ | Serious^4^ | Not serious | Serious^6^ | Serious^2^ | Very low | Hou, 2015^[14]^ |
| Qigong | Sham group; no intervention | Pain relieving | Serious^8^ | Serious^4^ | Not serious | Serious^6^ | Serious^2^ | Very low | Hou, 2015^[14]^ |
| Qigong | Sham group; no intervention | Function improving | Serious8 | Not serious | Not serious | Not serious | Serious^2^ | Low | Hou, 2015^[14]^ |
| Acupuncture | sham acupuncture | Pain relieving | Serious^9^ | Serious^4^ | Not serious | Not serious | Not serious | Low | Cao, 2012^[24]^ |
| Acupuncture | sham acupuncture | Function improving | Serious^9^ | Serious^4^ | Not serious | Not serious | Not serious | Low | Cao, 2012^[24]^ |
| Acupuncture | standard care | Pain relieving | Serious^10^ | Serious^4^ | Not serious | Not serious | Serious^11^ | Very low | Cao, 2012^[24]^ |
| Acupuncture | standard care | Function improving | Serious^10^ | Serious^4^ | Not serious | Not serious | Serious^11^ | Very low | Cao, 2012^[24]^ |
| Acupuncture | waiting list | Pain relieving | Serious^12^ | Serious^4^ | Not serious | Serious^6^ | Serious^11^ | Very low | Cao, 2012^[24]^ |
| Acupuncture | waiting list | Function improving | Serious^12^ | Serious^4^ | Not serious | Serious^6^ | Serious^11^ | Very low | Cao, 2012^[24]^ |
| Acupuncture | sham acupuncture | Pain relieving | Serious^13^ | Serious^4^ | Not serious | Not serious | Not serious | Low | Manheimer, 2007^[25]^ |
| Acupuncture | sham acupuncture | Function improving | Serious^13^ | Serious^4^ | Not serious | Not serious | Not serious | Low | Manheimer, 2007^[25]^ |
| Acupuncture | waiting list, standard care | Pain relieving | Serious^14^ | Serious^4^ | Not serious | Serious^6^ | Serious^11^ | Very low | Manheimer, 2007^[25]^ |
| Acupuncture | waiting list, standard care | Function improving | Serious^14^ | Not serious | Not serious | Serious^6^ | Serious^11^ | Very low | Manheimer, 2007^[25]^ |
| Acupuncture | sham acupuncture; no treatment | Less adverse effects | Not reported | Serious^4^ | Not serious | Not serious | Serious^2^ | Very low | Yamashita, 2006^[26]^ |
| Moxibustion | sham moxibustion; drug therapy | Quality of life (BP) | Not serious | Not serious | Not serious | Serious^15^ | Serious^11^ | Low | Li, 2016^[27]^ |
| Moxibustion | sham moxibustion; drug therapy | Function improving | Not serious | Very serious^4^ | Not serious | Serious^15^ | Serious^11^ | Very low | Li, 2016^[27]^ |
| Moxibustion | sham moxibustion; drug therapy | Pain relieving | Not serious | Very serious^4^ | Not serious | Serious^15^ | Serious^11^ | Very low | Li, 2016^[27]^ |
| Moxibustion | Intra-articular injection / oral drug | Response rate improving | Serious^16^ | Not serious | Not serious | Not serious | Serious^11^ | Low | Song, 2016^[28]^ |
| Moxibustion | oral drug, usual care, and sham moxibustion | Pain relieving | Serious^17^ | Serious^4^ | Not serious | Not serious | Serious^11^ | Very low | Song, 2016^[28]^ |
| Moxibustion | Drug therapy, usual care, and sham moxibustion | Function improving | Not serious | Serious^4^ | Not serious | Serious^15^ | Serious^11^ | Very low | Song, 2016^[28]^ |
| Duhuo Jisheng decoction plus glucosamine | glucosamine | Total WOMAC scores | Serious^18^ | Not serious | Not serious | Serious^15^ | Serious^11^ | Very low | Zhang,2016^[29]^ |
| Duhuo Jisheng decoction plus meloxicam and glucosamine | meloxicam and  glucosamine | Total WOMAC scores | Serious^19^ | Not serious | Not serious | Serious^15^ | Serious^11^ | Very low | Zhang,2016^[29]^ |
| MCHF | usual treatment | VAS-1 (global pain) / VAS-2 (pain on walking) / WOMAC score / Lysholm score | Serious^20^ | Serious^4^ | Not serious | Serious^15^ | Serious^11^ | Very low | Zhu, 2015^[30]^ |
| MCHF plus usual treatment | usual treatment | VAS-1 (global pain) / Lequesne index / Lysholm score | Serious^21^ | Serious^4^ | Not serious | Serious^15^ | Serious^11^ | Very low | Zhu, 2015^[30]^ |
| Chinese herbal bath therapy | Drug therapy | Pain relieving | Serious^22^ | Not serious | Not serious | Not serious | Serious^11^ | Low | Chen, 2015^[31]^ |
| Chinese herbal bath therapy | Drug therapy | overall effectiveness | Serious^23^ | Not serious | Not serious | Not serious | Serious^11^ | Low | Chen, 2015^[31]^ |
| Tai Chi | Education; usual care; no treatment | Pain relieving | Serious^24^ | Serious^4^ | Not serious | Serious^15^ | Serious^11^ | Very low | Ye, 2014^[32]^ |
| Tai Chi | Education; usual care; no treatment | Function improving | Serious^24^ | Serious^4^ | Not serious | Serious^15^ | Serious^11^ | Very low | Ye, 2014^[32]^ |

Footnotes: ^1^ We downgraded one level because lack of blinding of patients and providers in 4 of 12 studies; it was unclear if allocation was concealed in 4 studies; ^2^ The grey literature was not searched; ^3^ We downgraded one level because lack of randomization, blinding of patients and providers in 3 of 11 studies; it was unclear if allocation was concealed in 3 studies; ^4^ There was unexplained inconsistency that was supported by nonoverlapping confidence intervals, high I^2^ values, and statistically significant heterogeneity of effect estimates; ^5^ It was unclear if allocation was concealed or patients were blinded in 3 studies; ^6^ The sample size was smaller than optimal information size; ^7^ It was unclear if allocation was concealed or patients were blinded in 2 studies; ^8^ We downgraded one level because lack of blinding of patients and providers in 2 of 3 studies; it was unclear if allocation was concealed in 1 studies; ^9^ We downgraded one level because lack of blinding of patients and providers in 2 of 10 studies; it was unclear if allocation was concealed in 1 studies; ^10^ We downgraded one level because lack of blinding of patients and providers in 5 of 5 studies; ^11^ No reasonable method was used to evaluate post publication bias; ^12^ We downgraded one level because lack of blinding of patients and providers in 4 of 4 studies; ^13^ We downgraded one level because lack of blinding of patients and providers in 7 of 7 studies; ; it was unclear if allocation was concealed in 2 studies; ^14^ We downgraded one level because lack of blinding of patients in 2 of 7 studies; ^15^ Certainty in evidence lowered because of a small number of events leading to wide confidence intervals; ^16^ We downgraded one level because lack of randomization in 4 of 7 studies, lacking of blinding of patients and providers in 7 studies; it was unclear if allocation was concealed in 7 studies; ^17^ We downgraded one level because lack of randomization in 2 of 6 studies, lacking of blinding of patients and providers in 4 studies; it was unclear if allocation was concealed in 4 studies; ^18^ It was unclear if randomization or allocation was concealed in 2 of 3 studies; blinding of patients and providers was lacked in 3 studies; ^19^ It was unclear if randomization or allocation was concealed in 2 of 2 studies; blinding of patients and providers was lacked in 2 studies; ^20^ We downgraded one level because randomization was unclear in 12 of 16 studies, lacking of blinding of patients and providers in 14 studies; it was unclear if allocation was concealed in 16 studies; ^21^ We downgraded one level because randomization was unclear in 4 of 6 studies, lacking of blinding of patients and providers in 4 studies; it was unclear if allocation was concealed in 6 studies; ^22^ We downgraded one level because lack of blinding of patients and providers in 4 of 4 studies; it was unclear if allocation was concealed in 4 studies; ^23^ We downgraded one level because lack of blinding of patients and providers in 11 of 13 studies; it was unclear if allocation was concealed in 12 studies; ^24^ We downgraded one level because lack of blinding of patients and providers in 6 of 6 studies; it was unclear if allocation was concealed in 3 studies.
